# MicroRNA‐124: An emerging therapeutic target in cancer

**DOI:** 10.1002/cam4.2489

**Published:** 2019-08-06

**Authors:** Xinqi Jia, Xu Wang, Xiaorong Guo, Jingjing Ji, Ge Lou, Junjie Zhao, Wenjia Zhou, Mian Guo, Maomao Zhang, Chao Li, Sheng Tai, Shan Yu

**Affiliations:** ^1^ Department of Hepatopancreatobiliary Surgery The Second Affiliated Hospital of Harbin Medical University Harbin China; ^2^ Department of Neurology The Second Affiliated Hospital of Harbin Medical University Harbin China; ^3^ Department of Pathology The Second Affiliated Hospital of Harbin Medical University Harbin China; ^4^ Department of Neurosurgery the Second Affiliated Hospital of Harbin Medical University Harbin China; ^5^ Key Laboratory of Myocardial Ischemia Department of Cardiology the Second Affiliated Hospital of Harbin Medical University Harbin China; ^6^ Department of Orthopedics the Second Affiliated Hospital of Harbin Medical University Harbin China

**Keywords:** cancer, miR‐124, miRNAs, oncotherapy target

## Abstract

MicroRNAs (miRNAs) are noncoding single‐stranded RNAs, approximately 20‐24 nucleotides in length, known as powerful posttranscriptional regulators. miRNAs play important regulatory roles in cellular processes by changing messenger RNA expression and are widely involved in human diseases, including tumors. It has been reported in the literature that miRNAs have a precise role in cell proliferation, programmed cell death, differentiation, and expression of coding genes. MicroRNA‐124 (miR‐124) has reduced exparession in various human neoplasms and is believed to be related to the occurrence, development, and prognosis of malignant tumors. In our review, we focus on the specific molecular functions of miR‐124 and the downstream gene targets in major cancers, which provide preclinical evidence for the treatment of human cancer. Although some obstacles exist, miR‐124 is still attracting intensive research focus as a promising and effective anticancer weapon.

## Graphical abstract

miRNA‐124 is closely related to the cancer development and progression, and decreased in many cancer types. Increasing miRNA‐124 levels, inhibiting cancer cell proliferation, and affecting clinical outcome. miRNA‐124 has the potential to be a target for cancer treatment.

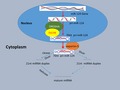

## 1 BACKGROUND

MicroRNAs (miRNAs or miRs) are endogenous noncoding RNA molecules, and single‐stranded RNAs composed of 20‐24 nucleotides participating in the regulation of gene translation.^1^ Previous studies have shown that miRNAs inhibit protein translation mainly by coupling with the base of the 3'‐untranslated region (3′‐UTR) of downstream coding‐gene mRNA.^2^ The expression of miRNAs is highly conserved in the process of biological evolution with tissue‐specific expression patterns in various cells. Accumulating evidence shows that the universality and diversity of miRNAs have a crucial role in cancer behaviors, such as carcinogenesis, angiogenesis, gluconeogenesis, and apoptosis, or through interaction with specific regulation targets that are essential for the development and progression of malignant tumors.^3^ In the present review, we will highlight recent studies on microRNA‐124 (hsa‐miR‐124) and the role it plays as a tumor suppressor gene in suppressing cancer cell proliferation and other cancer hallmarks.

## 2 INTRODUCTION TO MIR‐124

In 2002, miR‐124 was first discovered in mice.^4^ Subsequent research showed that miR‐124 is highly conserved and has been found to be expressed in both simple nematodes and complex humans. As a novel member of the noncoding RNA family, miR‐124 was found to be dysregulated in many malignant tumors. To date, three genes of human miR‐124 have been identified and located as follows: miR‐124a‐1(8p23.1), miR‐124a‐2(8q12.3), and miR‐124a‐3(20q13.33).^5^ Promoters at those locations all contain CpG islands.^6^ If the CpG island is highly methylated, the miR‐124 encoding gene is found to have a silencing effect,^7^ which further causes abnormal expression of miR‐124 and inactivation of the miR‐124 target mRNA.^8^ These regulatory networks result in biological effects for cancers.

**Figure 1:**
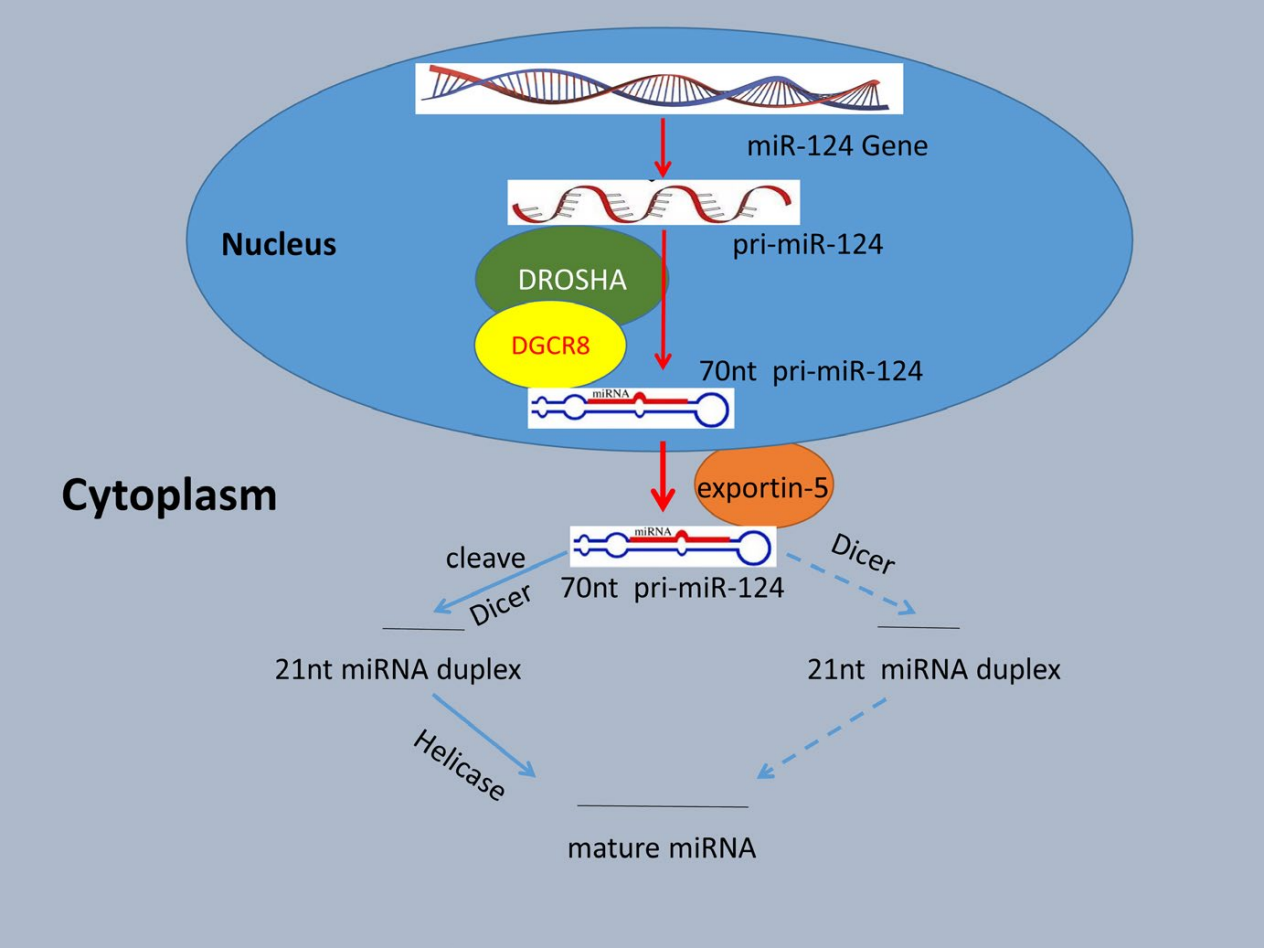
The precoding flow chart of mature miR‐124

The mature miR‐124 generation process is quite complex and has the following five steps (Figure 1): In the first step, the gene encoding miR‐124 is transcribed into primary miR‐124 (pri‐miR‐124) by RNA polymerase II in the cell nucleus; second, pri‐miR‐124 is shear processed via the Drosha‐Database of Gene Co‐Regulation 8 (DGCR8) complex into a precursor of miR‐124 (pre‐miR‐124) that is approximately 70 nucleotides (nt) long with stem‐loop structure. In the third step, pre‐miR‐124 is transported from the nucleus to the cytoplasm by the transporter exportin‐5; next, pre‐miR‐124 is cleaved by the nuclease Dicer into a double‐stranded RNA molecule of approximately 21 nt; finally, one strand of the double‐stranded RNA is degraded by helicase, and the other strand becomes a mature miRNA (known as miR‐124‐3p or 5p). Under the guidance of the RNA‐induced silencing complex (RISC), mature single‐stranded RNA can degrade or inhibit target gene translation by base pairing with 3'‐UTR of downstream coding RNAs in accordance with the principle of complete or incomplete base complementary pairing, thus playing a related regulatory role at the posttranscriptional level.

In our review, we mainly address the functions of miR‐124 in major cancers and its regulatory mechanism. In addition, we discuss that miR‐124 may be used as a therapeutic target and wide application prospects for cancer treatment potential.

## 3 BIOLOGICAL FUNCTIONS OF MIR‐124

As miRNAs have become a hot spot in the field of disease research, there is much evidence showing that miRNAs play a crucial role in a variety of biological processes.^9^ miR‐124 is abnormally expressed in inflammatory diseases (chronic sinusitis, ulcerative colitis) and immune disorders (rheumatoid arthritis) and acts as an inflammation response inhibitor.^10^, ^11^, ^12^ Moreover, it has been documented that miR‐124 has a wide range of biological effects and is involved in cell proliferation, autophagy, and neuronal differentiation.^13^, ^14^, ^15^ For instance, miR‐124‐3p has a significant neuroprotective function in Alzheimer's disease (AD) by targeting Caveolin‐1 directly. miR‐124‐3p mimics could reduce hyperphosphorylation of Tau protein and attenuate neuronal cell apoptosis, which is dependent on the Caveolin‐1‐PI3K/Akt/GSK3‐beta pathway.^16^ Increasing miRNA expression levels could be considered a novel prevention method for AD. Another report showed that miR‐124 could mediate the downregulation of HDAC5 and promote neurite development by activating the MEF2C‐GPM6A pathway in primary neurons.^17^

It has been reported that the reduction in miR‐124 levels in cancers is related to the decreased expression of cancer genes and related pathways.^18^, ^19^ However, studies have shown that miR‐124 may act as an inhibitor of the progression of oncogenes in tumors by regulating different target genes.^20^ For example, Zhao et al found that increasing miR‐124 expression in JHU‐22 cells could significantly inhibit cell proliferation, colony formation, and in vivo tumor diameter, showing a promising role for miR‐124 in tumor progression.^21^ Another report showed that Slit‐Robo GTPase‐activating protein 1 (SRGAP1) is a miR‐124 downstream‐regulated gene and that downregulation of miR‐124 in gastric cancer leads to overexpression of SRGAP1 and plays an oncogenic role.^22^ Nevertheless, more evidence demonstrated that miR‐124 has the main functions of inhibiting tumor growth, proliferation, and even chemotherapeutic sensitization.^23^, ^24^

## 4 MiR‐124 in Metabolism and Development

miRNAs play an important regulatory role in metabolism. Given the abundance of miR‐124 in the liver, it has been found to be directly involved in the regulation of cholesterol and liver fatty acid metabolism. As abnormal metabolism is a hallmark of cancer, miR‐124 was found to participate in fatty acid and triglyceride hydrolysis by suppressing the expression of several key enzymes in the mitochondrial β‐oxidation pathway, indicating its contribution to cancer proliferation and progression.^25^, ^26^ Moreover, insulin is known to be a powerful regulator of lipogenesis and lipolysis, and direct evidence found that miR‐124a was enriched in pancreatic tissue and involved in pancreas development in mouse embryos and in humans.^27^, ^28^ Baroukh et al found that miR‐124a is closely related to pancreatic cell differentiation and insulin secretion by targeting Rab27A and Noc2.^29^, ^30^ Overexpression of miR‐124a in mouse insulinoma cells (MIN6) inhibited the expression of FoxA2 and its downstream targets Pdx‐1, Kir‐6.2, and Sur‐1, suggesting that it also plays an important role in pancreatic insulin exocytosis.^30^ As miR‐124 is widely present in a variety of animals and is highly conserved during evolution, its vital role in tumorigenesis‐related metabolism cannot be neglected.

miR‐124 also acts as a critical factor for the regulation of neurodevelopment or neurogenesis in different species, including Drosophila, mouse, zebrafish, Xenopus, and human.^31^, ^32^, ^33^, ^34^, ^35^, ^36^ Related studies showed that overexpression of miR‐124 in the nervous system leads to abnormal development of Drosophila eyes, and after knocking out miR‐124, Drosophila has defects in all aspects, such as climbing, flight, development, and spawning ability.^31^ Kapsimali et al found that miR‐124 was involved in zebrafish embryo neuronal development and differentiation of the nerve cells.^37^

Another study reported that overexpressed miR‐124 could induce apoptosis of mouse embryonic neuronal carcinoma cells by targeting downstream signaling of Ezh2 genes.^32^ More data demonstrated that miR‐124 promoted neuronal differentiation of and neurite outgrowth in mouse inner ear neural stem cells (NSCs) by regulating tropomyosin receptor kinase B (TrkB) and cell division control protein 42 homolog (Cdc42).^34^ Another study also elucidated that miR‐124 promoted neural differentiation of NSCs by directly targeting DLL4 through suppression of the Notch pathway.^38^ Some scholars have suggested that Lhx2 is a primary target gene of miR‐124a and that Lhx2 downregulation by miR‐124a is required for the prevention of apoptosis in the developing retina and for proper axonal development of hippocampal neurons.^39^ Kaili Liu et al showed that NeuroD1 could act as a direct target of miR‐124 in vivo to control Xenopus cell proliferation and neurogenesis.^35^ The above evidence indicates that aberrant expression or function of miR‐124 could lead to diseases during development, especially in the nervous system.

## 5 MIR‐124 AND TUMORS

Malignant tumors are diseases caused by abnormal cell growth and proliferation. Modern medicine has recognized that cancer is defined as a kind of genetic mutation. miR‐124 is also an epigenetically silenced noncoding small RNA that was found to be decreased in a variety of malignant tumors,^40^ such as liver cancer, breast cancer, pancreatic cancer, and gastric cancer. Moreover, miR‐124 has been found to be expressed significantly differently in cancer tissues and adjacent tissues and is stably preserved in body fluids and tissues,^41^ suggesting that miR‐124 could be considered a biomarker and biological target for cancer diagnosis and treatment.

## 6 MIR‐124 AND HEPATOCELLULAR CARCINOMA

Patients with hepatocellular carcinoma (HCC) often have poor clinical outcomes due to distant metastasis and postoperative recurrence.^42^ Therefore, the potential mechanism of tumor growth and metastasis is critical in HCC treatment. Chen et al showed that aquaporin 3 (AQP3) was a downstream target of miR‐124. Increased AQP3 expression was found in HCC and was negatively correlated with miR‐124 expression. AQP3 could be reduced by overexpressing miR‐124, thereby inhibiting the proliferation and migration of hepatoma.^43^ Another HCC‐related study demonstrated that miR‐124 could further inhibit the proliferation of HCC cells (SMMC‐7721) by regulating Baculoviral IAP Repeat Containing 3 (BIRC3) and controlling the NF‐kB pathway and migration. The author also pointed out that miR‐124 was a therapeutic target for the prognosis of HCC.^44^ Other scholars have also pointed out that in two liver cancer cell lines (SMMC‐7721 and BEL‐7404), miR‐124 could regulate Sp1 expression and inhibit cell migration and invasion.^45^ Lu et al further confirmed that miR‐124 suppressed HepG‐2 cell proliferation by reducing STAT3 protein expression levels, and overexpressed miR‐124 could significantly promote HCC apoptosis.^46^ Another research group led by Lang et al showed that restoration of miR‐124 suppressed HCC proliferation and tumorigenesis by blocking G1 phase cells. They also found that phosphoinositide 3‐kinase catalytic subunit alpha (PIK3CA) is a downstream target of miR‐124 that plays a major role in HCC cell proliferation. Overexpression of miR‐124 could result in a decrease in PIK3CA expression and translation. As a negative regulator and inhibitor of tumor cytokines, miR‐124 achieves this by inhibiting the expression of PIK3CA and further influencing the PI3K/Akt pathway.^47^ In 2016, Xu et al found that in four liver cancer cell lines (MHCC‐LM3, Huh7, MHCC‐97L, and HepG2), miR‐124 downregulation was due to promoter hypermethylation, and a rescue experiment found that this miRNA could inhibit cancer susceptibility candidate gene 3 (CASC3) expression, inactivate the key molecules p38, JNK, and ERK in the mitogen‐activated protein kinases (MAPK) signaling pathway, and reduce the proliferation of HCC cells.^48^ Long et al confirmed that the expression of miR‐124‐3p was crucial for the development of HCC cell lines (HepG2 and Huh7). Clinical parameter association analysis showed that lower miR‐124‐3p was significantly correlated with tumor size, number, and clinical stage. Therefore, miR‐124‐3p can be used as a specific biomarker for patients with liver cancer.^49^ Moreover, LAMC1 miRNA response elements (MREs) could promote liver cancer progression by sponging miR‐124 and enhancing the expression of the miR‐124 downstream target CD151.^50^

It can be inferred that the abnormal expression of miR‐124 has an association with HCC progression. miR‐124 could be considered a potential tumor suppressor gene in liver malignancies. These findings show us new concepts that reveal the molecular network underlying liver cancer development and progression.

## 7 MIR‐124 AND BREAST CANCER

Breast cancer (BC) is the main cause of cancer‐related deaths among females globally.^51^ It is composed of various pathogenic factors, including genetic and epigenetic changes.^52^ The role of miRNAs in the occurrence and progression of BC has been gradually clarified. This finding suggested that gene silencing caused by methylation in the promoter of the miR‐124 encoding gene may be one reason for the decrease in miR‐124 in cancer cells with strong migration ability and may be a disadvantageous independent prognostic factor in BC patients.^53^ Li et al identified flotillin‐1 (FLOT1) as a direct downstream gene of miR‐124, and low miR‐124 expression restrained FLOT1 by acting on the 3'‐UTR of FLOT1, as confirmed by luciferase assays.^54^ They also found that miR‐124 was decreased in pathological BC tissues. In addition, Liang et al demonstrated that miR‐124 expression was lower than that in adjacent normal tissues in 37 of 38 breast neoplasms. Their findings identified Slug as a direct target gene of miR‐124. As a result, downregulation of miR‐124 increased the expression of E‐cadherin, which participates in Beta‐catenin/EMT‐related pathways. These findings suggested that miR‐124 plays an important role in BC invasion and metastasis.^55^ Zhang et al confirmed that Beclin‐1 could be regulated by miR‐124‐3p. Furthermore, they validated that miR‐124‐3p decreased in both BC samples and two ER‐positive cell lines (MDA‐MB‐468 and MCF‐7). They also pointed out that BC progression was dependent on the autophagy‐related protein Beclin‐1.^56^ Hu et al determined that the contributions of dual therapeutic targeting of miR‐124‐3p and ATP‐binding cassette subfamily C member 4 (ABCC4) in BC cells (MCF‐7‐ADR) may enhance drug sensitivity for Adriamycin‐resistant BCs. Moreover, western blot analysis showed that miR‐124‐3p overexpression significantly inhibited permeability glycoprotein 1/multidrug resistance protein 1 (P‐gp) expression in MCF‐7‐ADR cells.^57^ Some scholars have shown that ZEB2 is a miR‐124 direct target gene. When studied in tissues with triple‐negative breast cancer (TNBC), ZEB2 expression levels were upregulated, and miR‐124 expression levels were decreased accordingly. Therefore, miR‐124 may have a role as a bridge to control TNBC epithelial‐mesenchymal transition (EMT) progress.^58^ However, miR‐124 hypomethylation only shows a higher survival rate in BC patients over 35 years old, and it could be considered a poor prognosis biomarker for very young BC patients.^59^

This evidence implies that low expression of miR‐124 could promote the occurrence, development, and drug resistance of BC and that increasing the expression level of miR‐124 may inhibit the proliferation and EMT of BC.

## 8 MIR‐124 AND PANCREATIC CANCER

Pancreatic cancer is always diagnosed in late stage due to the lack of clinical manifestations.^60^ So far, there are not many effective targeted therapies to reduce its mortality.^61^ Emphasis has been placed on deeply investigating miR‐124 to reveal its role as a tumor suppressor or proto‐oncogene. Wang et al demonstrated that the miR‐124 genes were frequently methylated in pancreatic cancer samples compared with that of adjacent tissues using pyrosequencing analysis. Furthermore, they characterized Ras‐related C3 botulinum toxin substrate 1 (Rac1) as a direct target of miR‐124 through a luciferase assay. The decreased Rac1 expression level caused inactivation of the MKK4‐JNK‐c‐Jun pathway and inhibited pancreatic cancer proliferation, invasion, and metastasis. They also demonstrated that a state of gene silencing is caused by high levels of miR‐124 methylation in patients with pancreatic cancer, and low miR‐124 expression levels were correlated with a lower rate of survival.^62^ Other researchers found that MCT1 was more highly expressed in pancreatic cancer than in adjacent tissue and that miR‐124 could inhibit the efflux of lactic acid by directly binding to MCT1, leading to cell acidification, which in turn suppresses pancreatic cancer proliferation and migration.^63^ Idichi et al confirmed that integrin α3 (ITGA3) and integrin β1 (ITGB1) were also targets of miR‐124‐3p in pancreatic cancer cell lines (PANC‐1 and SW1990). miR‐124‐3p significantly inhibited invasion of the pancreatic cell lines by reducing those two target genes and impacting the PI3K/Akt pathway.^64^ In addition, EphA2 (erythropoietin‐producing hepatocellular receptor 2) was shown to be another regulatory downstream gene of miR‐124. As overexpressing EphA2 could promote drug resistance to erlotinib in BXPC‐3 cells, miR‐124 might function as a drug‐sensitive enhancer to improve pancreatic cancer patient clinical outcomes.^65^

These explanations demonstrate that miR‐124 has a strong association with the occurrence, progression, and prognosis of pancreatic cancer, suggesting that miR‐124 also plays a similar role as tumor suppressor genes in pancreatic cancer. However, different reports of miR‐124 are still emerging. The restoration of miR‐124 in vivo may be a dilemma for tumor therapy.

## 9 MIR‐124 AND GASTRIC CANCER

Gastric cancer (GC) is one of the main problems that endangers human health. Despite continuous medical advances, GC is still the third most prevalent tumor type in the world, leading to death.^66^ The inhibitory effect of miR‐124 on cancer growth and migration, as well as the dysregulation of miR‐124, has been reported in GC.^67^, ^68^ For example, in 2012, Xia et al showed that miR‐124 suppressed the proliferation of GC cells and reduced the carcinogenicity of GC by binding to the target gene SPHK1, implying that miR‐124 plays an important role in the occurrence and progression of GC through the AKT‐FOXO1‐p21^Cip1^‐p27^Kip1^ signaling pathway.^69^ Hu et al also confirmed that miR‐124 had the ability to inhibit GC cell (SGC‐7901) proliferation, migration, and invasion by inhibiting Rho‐associated protein kinase 1 (ROCK1).^70^ Later, in 2014, Xie et al revealed that upregulating miR‐124 levels not only suppressed proliferation but also promoted apoptosis in GC cell lines (MKN‐74, MKN‐28, and MKN‐45). They found that miR‐124 could target the Jagged1 (JAG1) gene and suppress the Notch signaling pathway, thus inhibiting the progression of GC.^71^ Other data supported SPHK1 as a direct target gene of miR‐124. Suppression of miR‐124 expression could inhibit GC proliferation and invasion via binding with the SPHK1 3'‐UTR in MGC‐803 cells.^72^ Liu et al found that miR‐124‐3p was involved in the carcinogenesis of GC (SGC‐7901 and MKN‐28) by dual‐targeting Rac1 and Sp1.^73^ Mirnoori et al first announced that the overexpression of pri‐miR‐124‐1 caused STAT3 mRNA level reduction and STAT3 phosphorylation. The effect of single‐nucleotide polymorphisms (SNPs) in miR‐124 strongly impacts GC susceptibility.^18^ Moreover, Zare et al identified that the RUNX2 and DDX6 genes are miR‐124 regulatory genes and that downregulation of miR‐124 could be a significant indicator of neoplastic transformation in GC cells.^67^

The above findings show that miR‐124 is involved in the occurrence and development of GC and provide experimental evidence for miR‐124 as a possible therapeutic target for GC.

## 10 MIR‐124 AND COLORECTAL CANCER

Colorectal cancer (CRC) is the main cause of cancer‐related death 74 worldwide. The incidence has kept rising in recent years. Therefore, in‐depth studies of the pathogenesis of CRC play an important role in human health. Liu et al demonstrated that underexpressed miR‐124 could regulate inhibitor of apoptosis‐stimulating protein of p53 (iASPP) in CRC patients and was related to the proliferation of CRC cells via influencing the iASPP/NF‐kB pathway.^60^ In addition, miR‐124 inhibited the target gene polypyrimidine tract‐binding protein 1 (PTB1) in the DLD‐1 CRC cell line. PTB1 is known to induce autophagy and apoptosis in CRC cells. This research supported the anticancer effect of miR‐124.^75^ Another report further showed that miR‐124 could reduce ROCK1 expression in CRC by western blot and then regulate the biological behavior of CRC cell line (SW620) hyperplasia.^76^ Zhang et al applied quantitative RT‐PCR to analyze the tissues of 90 patients who underwent CRC surgery. Their results confirmed that miR‐124 was lower in CRC tumor samples compared with that in normal tissue. At the same time, overexpressed miR‐124 could induce apoptosis of CRC cells and inhibit the growth of tumors by targeting STAT3 and regulating its downstream signaling.^77^ In addition, miR‐124 could combine with the 3'‐UTR of the IQ motif containing GTPase‐activating protein 1 (IQGAP1). A miR‐124 mimic could inhibit IQGAP1 protein levels in CRC, thereby inhibiting CRC proliferation.^78^ Another study found that in doxorubicin (DOX)‐resistant CRC tumors and cell lines (HCT116 and LoVo, respectively), the lncRNA X‐inactive specific transcript (XIST) was increased and negatively correlated with the expression of miR‐124. They also showed that the downstream target gene of miR‐124 was SGK1. In addition, their findings suggested that XIST and miR‐124 may be ideal therapeutic targets to promote the DOX‐based chemotherapy effect in CRC.^65^

Briefly, miR‐124 exerts a tumor suppressor role by targeting its downstream genes during the development of CRC. In particular, miR‐124 could serve as a powerful diagnostic and therapeutic tool for CRC.

## 11 MIR‐124 AND BRAIN TUMORS

Originally cloned from mouse brain tissue, miR‐124 is highly conserved in animals and is the most abundantly expressed miRNA in the embryonic and adult brain, serving a key role in neurogenesis.^79^ miR‐124 is highly expressed in differentiated and mature neurons but has low expression in NSCs and glial cells.^80^ A few studies have shown that miR‐124 is significantly downregulated in glioblastoma and medulloblastoma and could be targeted for the treatment of these conditions.^81^, ^82^

Related literature revealed that self‐renewal and tumorigenic ability of human glioblastoma cells are achieved by miR‐124 via inhibiting small C‐terminal domain phosphatase 1 (SCP1).^83^ Another research group also confirmed the inhibitory role of miR‐124 in glioblastoma cells through the reduction in ROCK1.^84^ Furthermore, abnormal miR‐124 expression levels could cause a remarkable suppression of glioblastoma migration and invasion, and the transfection of a miR‐124 mimic into glioblastoma led to the stagnation of glioblastoma cells at the G1 phase.^85^ Moreover, the binding of miR‐124‐3p to the 3'‐UTR of STAT3 inhibited the protein expression of STAT3 in glioblastoma cell lines (U87 and U251), indicating another therapeutic target for glioblastoma patients.^86^, ^87^ Wang et al found that the circular RNA MMP9 (circMMP9) gene could sponge miR‐124, which is a novel underlying mechanism in glioblastoma cells (U87 and U251), regulating glioblastoma multiforme cell tumorigenesis through the circMMP9/miR‐124‐CDK4/AURKA signaling axis.^88^ Luo et al found that miR‐124‐3p could decrease Fra‐2 mRNA levels by a dual‐luciferase reporter assay, and increasing miR‐124‐3p in glioma cells could prevent classic cancer biological behaviors.^89^ Moreover, Wu et al validated that miR‐124‐3p could inhibit EphA2 expression and its downstream pathway to regulate the growth and invasion of glioma cells (U87MG and LN229).^90^ Yang et al confirmed that overexpression of miR‐124‐3p directly inhibited lncRNA homeobox (HOX) A11‐AS expression in glioma cells using a luciferase assay, indicating the importance of the lncRNA HOXA11‐AS/miR‐124‐3p axis in the regulation of glioma progression.^91^ A clinical study showed that plasma miR‐124 expression in glioma patients was significantly downregulated compared to normal healthy controls, suggesting that plasma miR‐124 could serve as an important biomarker for glioma prognosis.^92^ Medulloblastoma is the most common primary cranial malignancy in children.^93^ Scientists compared the expression levels of miR‐124 in normal cerebellar tissue and medulloblastoma and found that miR‐124 was significantly lower in medulloblastoma. After increasing miR‐124 in medulloblastoma cells (D425), cell proliferation was inhibited. This phenomenon was important based on the interaction between miR‐124 and CDK6.^94^ Some researchers used real‐time PCR to measure the expression levels of growth‐associated protein 43 (GAP‐43), calreticulin (CRT), and neuron‐specific enolase (NSE), and their results showed that miR‐124 exerted diverse functions and controlled multiple targets in the differentiation process of malignant brain tumors (SK‐N‐SH cell line).^95^

These studies emphasize the crucial role of miR‐124 in the regulation of brain tumor biological behavior and indicate that miR‐124 is a promising and novel therapeutic target for glioblastoma invasion.

## 12 MIR‐124 AND CERVICAL CANCER

Cervical cancer has become the most common gynecologic malignancy due to widespread human papillomavirus (HPV) infection.^96^ Abnormal miR‐124 expression due to promoter methylation is functionally involved in cervical cancer. The miR‐124 promoter region containing CpG islands in normal cells is usually hypomethylated or unmethylated. A report stated that methylation of miR‐124 was found in the cells that detected in cervical cancer, suggesting that miR‐124 gene silencing caused by hypermethylation may play a role in cervical cancer formation. As the miR‐124 level in cervical cancer tissues is significantly lower than in normal tissues, overexpression of miR‐124 in HPV‐positive cell lines (SiHa and CaSki) could significantly suppress cell proliferation and migration by regulating the IGFBP7 gene.^97^ Another HPV‐related study suggested that LMX1A gene methylation and the miR‐124‐2 gene were predictors of the presence of high‐grade lesions, regardless of virus infection.^98^ The latest findings showed that miR‐124‐3p could suppress the proliferation and metastasis of cervical cancer by inhibiting the expression of insulin‐like growth factor 2 mRNA‐binding protein 1 (IGF2BP).^99^

Many earlier studies have shown that miR‐124 is associated with cervical cancer tumorigenesis and acts as a tumor suppressor gene. Thus, the underlying molecular mechanism of miR‐124 is worth studying to reveal its nature in cervical cancer.

## 13 MIR‐124 AND PROSTATE CANCER

Prostate cancer (PC) is a common malignant tumor in older men 100 and is one of the most fatal human cancers due to its insidious onset and the current lack of robust early diagnostic tests. miR‐124 is an independent prognostic marker for PC.^88^ The androgen receptor (AR) is known to have a strong association with the initiation and progression of PC. miR‐124 was found to inhibit PC cell growth by targeting AR and increasing p53 expression levels.^20^ Some scientists analyzed the expression of miR‐124 in normal prostate cells and PC cells, and an RT‐PCR reporter assay showed that the expression of miR‐124 in PC cells was low, suggesting that reduction in miR‐124 may be related to the occurrence and development of PC.^101^ Investigators transfected miR‐124 into PC cells and showed significant inhibition of cancer cell proliferation, providing additional evidence for the role of miR‐124 as a tumor suppressor gene.^102^ These findings support that miR‐124 has a theoretical basis for gene therapy in PC.^103^

Findings on miR‐124 show that it may act as a therapeutic target gene and improve survival through delayed development of PC.

## 14 MIR‐124 AND OTHER MALIGNANT TUMORS

With the gradual deepening of research on regulation of miR‐124 gene expression, the abnormal expression of miR‐124 in lung cancer, nasopharyngeal carcinoma, cholangiocarcinoma, gallbladder cancer, ovarian cancer, bladder cancer, renal carcinoma, retinoblastoma, and acute lymphoblastic leukemia has been discovered. miR‐124 can bind to the 3'‐UTR of CDH2 to inhibit nonsmall cell lung cancer (NSCLC) cell proliferation invasion.^104^ Li et al suggested that lncRNA 1308 may function as a competing endogenous RNA (ceRNA) for miR‐124 to regulate lung cancer cell invasion through the miR‐124/ADAM 15 (a disintegrin and a metalloproteinase 15) signaling pathway, indicating that lncRNA 1308 and miR‐124 both have important roles in the disease progression of NSCLC.^105^ Tang et al also demonstrated that long noncoding RNA (OGFRP1) could sponge miR‐124‐3p and work as a ceRNA to increase the miR‐124 downstream target gene LYPD3, implying that OGFRP1 and miR‐124‐3p could be novel therapeutic targets for lung cancer.^106^ Xiao et al found that miR‐124 was significantly decreased in nasopharyngeal carcinoma (NPC) and that a miR‐124 mimic could inhibit NPC cell (CNE1 and CNE2) proliferation, migration, and invasion via binding with the target gene forkhead box Q1 (Foxq1).^107^ In a recent study, miR‐124 inhibited TGF‐β‐induced cell proliferation and migration through SMAD and ERK‐related signaling pathways in NPC cells.^108^ Other research on cholangiocarcinoma (CCA) showed that miR‐124 could inhibit cell invasion and metastasis by targeting GATA6.^109^ In regard to gallbladder carcinoma, studies showed that miR‐124 had a decreased expression level in clinical samples and cell lines (QBC939 and GBC‐SD). These results also confirmed that circHIPK3 is also a target of miR‐124, which makes miR‐124 a new therapeutic target for the treatment of gallbladder carcinoma.^110^ Zhang et al found that miR‐124 was downregulated in ovarian cancer cell lines (skov3‐ip and ho8910pm) and tumor tissues. They also suggested that SphK1 (Sphingosine kinase 1) is a target of miR‐124 in ovarian cancer. Thus, it is possible that miR‐124 could attenuate ovarian cancer invasion partly through inhibition of the SphK1 pathway.^111^ A report stated that the expression of miR‐124 could inhibit the growth of human epithelial ovarian cancer cell xenografts.^112^ Wang et al demonstrated that miR‐124 could regulate STAT3 protein expression and have a tumor suppressor function in proliferation and apoptosis in bladder cancer cell lines (T24) through the miR‐124/STAT3 signaling pathway.^113^ Another study on the same bladder cancer cell line showed that miR‐124 controlled the target gene Caveolin 1 (CAV1) and played a suppressive role in the proliferation, migration, and invasion of bladder cancer.^114^ Additionally, the lncRNA MALAT1 may function as a ceRNA to sponge miR‐124 by modulating the depression of foxq1 at posttranscriptional levels and promoting bladder transitional cell carcinoma cell (BIU‐87) proliferation, migration, and invasion.^115^ Zo et al also verified that miR‐124 had the ability to inhibit proliferation, migration, and invasion of bladder cancer by regulating DNA methyltransferase 3B (DNMT3B).^116^ Wang et al reported that STAT3 and matrix metalloproteinase‐9 (MMP‐9) were also direct targets of miR‐124 in renal carcinoma, regulating renal cancer cell OS‐RC‐2 invasion ability.^117^ Additional evidence demonstrated that the lncRNA HOTAIR regulates miR‐124 in renal carcinoma cell lines (ST8SIA4). Overexpression of lncRNA HOTAIR promoted proliferation, migration, and invasion in renal carcinoma cells.^118^ Hu et al revealed that STAT3, as a signal transducer and activator, is a downstream gene in the miR‐124 regulatory network in retinoblastoma and that miR‐124 expression is controlled by the lncRNA XIST. The interaction between XIST and miR‐124/STAT3 contributed to the progression of retinoblastoma.^119^ Finally, epigenetic regulation of small nucleolar RNA host gene 16 (SNHG16) was also a target of miR‐124‐3p, and inhibiting miR‐124‐3p reversed SNHG16‐mediated tumor suppressive functions in acute lymphoblastic leukemia (ALL).^120^

## 15 CONCLUSION AND FUTURE PERSPECTIVE

The abnormal expression of miR‐124 in tumors suggests that it can be used as an important target for the early diagnosis, treatment, and prognosis of tumors, which is conducive to the development of targeted molecular therapy and individualized treatment using miRNA tools. The role of miR‐124 in the overall molecular network of organisms still needs to be further explored. At present, the primary task of researchers is exploring the mechanisms of miR‐124 in the physiological and pathological states (Table 1). We believe that further studies on the regulatory functions and mechanisms of miR‐124 in animal genes will bring more benefits to the treatment of human cancers. A deeper and broader study remains to determine the role of miR‐124 in different cancers. However, certain challenges are emerging along with clinical in vivo application. One of the major problems is miRNA degradation and immunoreaction. Concentration and length of time at the target area are also obstacles. Although nanocarriers may help to solve these problems, the toxicity and unexpected side effects of nanoparticles are worthy of attention. The off‐target effect and accumulation in healthy tissues are still an area for further study. Even so, solid evidence continues to emerge, showing that the restoration of miR‐124 expression could be a promising therapeutic strategy for cancer treatment.

**Table 1:** Current targets and functions of microRNA‐124 in malignant tumors

Cancer types	Target gene(s)	Biology functions	Reference
Hepatocellular carcinoma	AQP3	Inhibit proliferation	43
	BIRC3	Inhibit proliferation	44
	Sp1	Inhibit migration	45
	STAT3	Inhibit proliferation	46
	PIK3CA	Inhibit proliferation	47
	CASC3	Inhibit proliferation	48
Breast cancer	FLOT1	Inhibit translation	54
	Slug	Inhibit invasion	55
	Beclin‐1	Promote proliferation	56
	ABCC4	Enhance drug sensitivity	57
	ZEB2	Inhibit proliferation	58
Pancreatic cancer	Rac1	Inhibit proliferation	62
	MCT1	Inhibit proliferation	63
	ITGA3, ITGB1	Inhibit proliferation	64
	EphA2	Enhance drug sensitivity	65
Gastric cancer	SPHK1	Inhibit proliferation	69, 72
	ROCK1	Inhibit proliferation	70
	JAG1	Inhibit proliferation	71
	Rac1, Sp1	Inhibit proliferation	73
	STAT3	Inhibit proliferation	18
	RUNX2, DDX6	Inhibit proliferation	67
Colorectal cancer	PTB1	Inhibit proliferation	75
	ROCK1	Inhibit proliferation	76
	STAT3	Inhibit proliferation	77
	XIST	Enhance drug sensitivity	65
Brain tumors	SCP1	Inhibit proliferation	83
	ROCK1	Inhibit proliferation	84
	STAT3	Inhibit proliferation	86, 87
	circMMP9	Inhibit proliferation	88
	Fra‐2	Inhibit proliferation	89
	EphA2	Inhibit proliferation	90
	CDK6	Inhibit proliferation	94
Cervical cancer	IGFBP7	Inhibit proliferation	97
	LMX1A	Inhibit proliferation	98
	IGF2BP	Inhibit proliferation	99
Prostate cancer	AR	Inhibit proliferation	20
Lung cancer	LYPD3	Inhibit proliferation	106
Nasopharyngeal carcinoma	Foxq1	Inhibit proliferation	107
Cholangiocarcinoma	GATA6	Inhibit metastasis	109
Ovarian cancer	SphK1	Inhibit metastasis	110
Bladder cancer	STAT3	Inhibit proliferation	113
	CAV1	Inhibit proliferation	114
	Foxq1	Promote proliferation	115
Renal carcinoma	STAT3, MMP‐9	Inhibit invasion	117
Retinoblastoma	STAT3	Promote proliferation	119
Acute lymphoblastic leukemia	SNHG16	Inhibit proliferation	120

Abbreviations: ABCC4, ATP‐binding cassette subfamily C member 4; AQP3, aquaporin 3; AR, androgen receptor; CASC3, candidate genes 3; CAV1, Caveolin‐1; EphA2, erythropoietin‐producing hepatocellular receptor 2; FLOT1, flotillin‐1; Foxq1, forkhead box Q1; IGF2BP, insulin‐like growth factor 2 mRNA‐binding protein 1; ITGA3, integrin α3; ITGB1, integrin β1; JAG1, Jagged1; MMP‐9, matrix metalloproteinase‐9; PIK3CA, phosphoinositide 3‐kinase catalytic subunit alpha; PTB1, polypyrimidine tract‐binding protein 1; Rac1, Ras‐related C3 botulinum toxin substrate 1; ROCK1, Rho‐associated protein kinase 1; SCP1, small C‐terminal domain phosphatase 1; SNHG16, small nucleolar RNA host gene 16; SP1, specificity protein 1; SphK1, sphingosine kinase 1; XIST, X‐inactive specific transcript.

## CONFLICT OF INTERESTS

The authors declare that they have no competing interests.

## AUTHOR CONTRIBUTIONS

All the authors contributed to the preparation of this manuscript. XQJ and XW were responsible for the literature search and the first draft of this article. XRG, GL, and JJJ were responsible for language polishing. JJZ and WJZ were drawing the figure. MG and MMZ contributed to further editing the manuscript. ST and SY revised the manuscript and were responsible for the concept design. All authors read and approved the final manuscript.
